# Outcomes and Risk Factors in Prosthetic Joint Infections by multidrug-resistant Gram-negative Bacteria: A Retrospective Cohort Study

**DOI:** 10.3390/antibiotics10030340

**Published:** 2021-03-23

**Authors:** Raquel Bandeira da Silva, Mauro José Salles

**Affiliations:** 1 Department of Internal Medicine, Hospital São Francisco de Assis, Belo Horizonte 30360-290, Brazil; girassoisnojardim@gmail.com; 2 Division of Infectious Diseases, Santa Casa de São Paulo School of Medical Sciences, São Paulo 01221-020, Brazil; 3 Laboratório LEMC, Disciplina de Infectologia, Departamento de Medicina, Universidade Federal de São Paulo-Escola Paulista de Medicina (UNIFESP-EPM), São Paulo 04025-010, Brazil

**Keywords:** surgical site infection, prosthetic joint infection, epidemiology, risk factors, multidrug-resistant Gram-negative bacteria, extensively drug-resistant, hematoma

## Abstract

Gram-negative bacteria (GNB), including multidrug-resistant (MDR) pathogens, are gaining importance in the aetiology of prosthetic joint infection (PJI). This retrospective observational study identified independent risk factors (RFs) associated with MDR-GNB PJI and their influence on treatment outcomes. We assessed MDR bacteria causing hip and knee PJIs diagnosed at a Brazilian tertiary hospital from January 2014 to July 2018. RFs associated with MDR-GNB PJI were estimated by bivariate and multivariate analyses using prevalence ratios (PRs) with significance at *p* < 0.05. Kaplan–Meier analysis was performed to evaluate treatment outcomes. Overall, 98 PJI patients were analysed, including 56 with MDR-GNB and 42 with other bacteria. Independent RFs associated with MDR-GNB PJI were revision arthroplasty (*p* = 0.002), postoperative hematoma (*p* < 0.001), previous orthopaedic infection (*p* = 0.002) and early infection (*p* = 0.001). Extensively drug-resistant GNB (*p* = 0.044) and comorbidities (*p* = 0.044) were independently associated with MDR-GNB PJI treatment failure. In sum, MDR-GNB PJI was independently associated with previous orthopaedic surgery, postoperative local complications and pre-existing infections and was possibly related to selective pressure on bacterial skin colonisation by antibiotics prescribed for early PJI. Infections due to MDR-GNB and comorbidities were associated with higher treatment failure rates.

## 1. Introduction

Joint replacement or arthroplasty aims to improve the mobility and quality of life of patients who experience painful symptoms or functional disability. However, prosthetic joint infection (PJI) is among the most feared complications that may result from such procedures, with an incidence of 1% to 2% among primary [1,2] and up to 4% among revision arthroplasties [3,4], respectively. Older patient age, revision arthroplasty, diabetes mellitus, rheumatoid arthritis, smoking, obesity and a high American Society of Anaesthesiologists (ASA) score are viewed as independent risk factors (RFs) for PJI [5,6,7,8,9].

Gram-positive cocci (GPC), such as *Staphylococcus aureus* and coagulase-negative staphylococci remain the primary etiological agents of PJI, with Gram-negative bacteria (GNB) identified less frequently [10]. Although few multicentre studies have described the microbiological epidemiology of PJI, the role played by GNB appears to be increasing. Rates of infections involving these organisms have ranged from 5% to 23% in previous investigations [4,7,10] but rates of greater than 40% have been reported for GNB-associated PJI in total knee arthroplasty (TKA) [11] and shoulder arthroplasty [6]. 

Due to the current scarcity of antibiotics able to control bone and biofilm infections, the emergence of MDR-GNB PJIs has become a growing concern in countries reporting high prevalence rates of MDR-GNB nosocomial infections, including postoperative infections [12,13]. Benito et al. [12] identified an increase in the prevalence of MDR-GNB arthroplasty infections from 5.3% between 2003 and 2004 to 8.1% between 2011 and 2012, with a corresponding increase in identified MDR-GNB strains, such as *Escherichia coli*, *Klebsiella pneumoniae*, *Pseudomonas aeruginosa* and *Morganella morgannii*. In the same study, a worrying increase in the rates of quinolone-resistant MDR-GNB strains was identified; quinolones represent an important and effective class of antibiotics often used to treat PJIs. Fantoni et al. [13], in a multicentre study of GNB-associated PJI, identified higher rates of MDR strains (53.7%), including 13.5% that expressed resistance to carbapenems, which are considered the last-line antibiotics for GNB infections.

Although PJIs caused by GNB appear to be increasing in frequency, current literature describing the epidemiology of PJI caused by MDR-GNB remains scarce, and few studies to date have attempted to investigate the outcomes and RFs associated with MDR-GNB PJI. Herein we describe a cohort of patients presenting with MDR-GNB PJIs and identify the predisposing independent factors associated with PJI caused by MDR-GNB and their influence on treatment outcomes.

## 2. Materials and Methods

### 2.1. Study Design

This was a retrospective, single-centre cohort study involving the identification and analysis of information from patient records describing hip and knee PJIs caused by MDR-GNB between January 2014 and July 2018 at an orthopaedic referral hospital centre. 

The primary study endpoint was the identification of independent predisposing factors associated with PJI caused by MDR-GNB. The secondary endpoint was the identification of independent variables influencing the treatment outcome of patients with MDR-GNB PJI. The study included individuals aged at least 18 years old who (a) met the criteria for arthroplasty infection as defined by the Musculoskeletal Infection Society (MSIS) [14] (Appendix A); (b) had at least one phenotypically indistinguishable aetiological agent that was identified in two or more samples of representative biological specimens; and (c) had at least one year of prospective follow-up data. Patients who underwent arthroplasty at an institution other than ours, had a follow-up period shorter than 12 months, did not meet the criteria for PJI as defined by the MSIS or had culture-negative results were excluded. Patients were selected from the infection database of the hospital infection control (IC) unit using surgical-site infection (SSI) notifications. Based on SSI notifications, patients’ medical records and results of microbiological cultures were located in specific databases to determine whether each patient fulfilled the inclusion criteria. The study was reviewed and approved by the local ethics committee (approval no. 2,610,914) on 20 April 2018.

### 2.2. Definitions

The PJI onset date was defined according to the date of the first observation of typical infectious signs and symptoms. For the purposes of this study’s analysis, only aetiological agents identified during the first debridement surgery were considered in cases subjected to multiple debridements. MDR-GNB was defined as the nonsusceptibility of the identified pathogen to at least one antimicrobial agent from three or more different antimicrobial classes (e.g., aminoglycosides, cephalosporins with an anti-*Pseudomonas* effect, carbapenems, fluoroquinolones, penicillin + β-lactamase inhibitors, monobactams and polymyxin). GNB that were extensively drug-resistant (XDR) to multiple antibiotics were defined as those lacking susceptibility to at least one antimicrobial agent from all but two classes of antimicrobials [15]. Early infections were defined as those with onset occurring less than three months after prosthesis placement. Long-term remission of PJI following treatment was defined as the absence of clinical, laboratory and radiological symptoms of infection at the last medical follow-up (with a minimum follow-up point of one year). Therapeutic failure was defined as infection recurrence at a previously controlled site; requirement for new surgery, a second course of antimicrobial therapy, chronic antibiotic suppression, excision arthroplasty or limb amputation, or death within the follow-up period [16,17].

### 2.3. Investigated Variables

To identify potential RFs associated with PJI caused by MDR-GNB as well as treatment outcomes for such infections, variables were obtained from patients’ medical records and surgical description sheets were reviewed. The potential variables reviewed for association with MDR-GNB PJI were categorised into three distinct groups as follows: (a) variables related to the patient, (b) variables related to the surgical procedure and (c) variables related to the postoperative period. The patient-related variables included demographics, comorbidities, alcoholism, smoking habits, ASA Physical Status Classification score, previous use of antimicrobials in the last three months and previous orthopaedic infection. The variables related to the surgical procedure were arthroplasty joint location, total or partial arthroplasty, revision surgery and post-trauma or elective arthroplasty. The variables related to the postoperative period were a concomitant infection during the same hospitalisation, the presence of postoperative hematoma, the presence of sepsis at the time of infection diagnosis and early or late infection. Operative variables such as debridement and implant retention (DAIR) or any prosthesis exchange used for the treatment of PJI were assessed when RFs for MDR-GNB PJI were considered in the outcomes analysis.

### 2.4. Microbiological Analysis

The institutional microbiological protocol consisted of synovial fluid (aseptically inoculated into standard aerobic blood culture bottles) and tissue sample analyses. Tissues obtained from the surgical procedure were homogenised in 3 mL of brain–heart infusion broth for one minute and inoculated onto aerobic sheep blood agar, chocolate agar and anaerobic blood agar and into thioglycolate broth (BD Diagnostic Systems, Hunt Valley, MD, USA). The time limit for processing samples was six hours. Aerobic plates were incubated aerobically at 35 °C to 37 °C in 5% to 7% CO_2_ for seven days, and anaerobic plates were anaerobically cultured at 37 °C for 14 days. Additionally, 0.5 mL of tissue homogenate was inoculated in thioglycolate broth for 14 days, and the turbid thioglycolate broth was subcultured on blood agar plates when cloudy. Colonies of microorganisms observed to be growing on the plates were identified, and their susceptibility to different antibiotics was tested according to standard microbiologic techniques [18]. The bacteria were identified by conventional biochemical and metabolic tests according to international standards and the definitions of the European Committee on Antimicrobial Susceptibility Testing (EUCAST) [18]. Sensitivity tests were performed using the disk-diffusion technique. If a minimum inhibitory concentration determination was necessary, automated or electronic test methods were used; the results are presented according to the EUCAST criteria that were valid at the time of testing [18].

### 2.5. Statistical Analysis

Qualitative variables for the overall study sample and the groups designated as infected by MDR-GNB and other bacteria, respectively, are described using mean and percentage values. Quantitative variables are described as using mean and standard deviation (SD), or median and interquartile range according to their observed distribution. Associations between qualitative variables were determined using the chi-squared test and Fisher’s exact test, and comparisons of means between groups using interval-type variables were performed using the Student’s t-test. Poisson regression was used to calculate prevalence ratios (PRs), using independent variables with significance levels below 25% (*p* < 0.25). Only those variables with a significance level below 5% (*p* < 0.05) were retained in the final model. To identify the variables related to treatment failure, Kaplan–Meier curves were constructed for each factor, and the log-rank test was used to compare the curves. Cox regression was used to identify predictor variables that influenced patient outcomes. All results were considered significant at a significance probability below 5% (*p* < 0.05). All data were analysed using the Statistical Package for the Social Sciences, version 23 (IBM Corporation, Armonk, NY, USA).

## 3. Results

### 3.1. Study Population

Overall, a total of 2672 arthroplasties were performed during the study period and a total of 115 PJI cases were assessed for inclusion in the study. Of these, 14 PJI cases that did not meet the MSIS criteria for infection and three PJI cases with negative cultures were excluded. Therefore, 98 PJI cases were analysed, including 56 (57.1%) and 42 (42.9%) caused by MDR-GNB and other microorganisms, respectively. 

The demographic, clinical features, comorbidities, surgical procedures and postoperative characteristics of the study population are summarised in Table 1. The mean age in the study population was 67 years (SD: ± 13.2 years), and 58.2% of the patients were female. Perioperative risk assessment varied, with 21.4% of cases classified as ASA 1, 48% as ASA 2 and 30.6% as ASA 3 or 4, respectively. More than 70% of the patients had at least one comorbidity. Hip arthroplasty was the most frequent procedure (83.7%), while 39.8% of the patients underwent arthroplasty due to trauma.

### 3.2. Microbial Identification

Overall, microbiological analysis yielded 104 microorganisms from 98 PJI patients. MDR-GNB was isolated from 30 patients (30.6%) and XDR-GNB from 26 (26.5%). The most prevalent pathogen was *Acinetobacter baumannii* (31.6%), followed by *S. aureus* among which 15.4% (16/104) were sensitive to methicillin (MSSA), and 4.8% (5/104) were methicillin-resistant (MRSA). Among patients with PJI caused by MDR or XDR-GNB, *A. baumannii* followed by *Enterobacter aerogenes*, *K. pneumoniae* and *E. coli* were the most commonly identified etiological agents. Microorganisms isolated from bone and soft tissue cultures of the 98 PJI patients included in this study are summarised in Table 2.

### 3.3. Potential Predisposing Factors for PJI Caused by MDR-GNB and Clinical Outcomes

As compared with PJIs caused by other microorganisms, infections due to MDR- and XDR-GNB in the univariate analyses were significantly associated with male sex (70.7% vs. 29.3%; *p* = 0.021), revision arthroplasty (66.1% vs. 11.9%; *p* = 0.000), metabolic syndrome (10.7% vs. 28.6%; *p* = 0.024), alcoholism (21.4% vs. 4.8%; *p* = 0.020), nonelective arthroplasty (55.4% vs. 19.0%; *p* = 0.000), previous use of antibiotics in the last three months (55.4% vs. 14.3%; *p* = 0.000), concomitant non-orthopaedic infection (19.6% vs. 4.8%; *p* = 0.000), previous orthopaedic infection (35.7% vs. 0%; *p* = 0.000), postoperative hematoma (51.8% vs. 2.4%; *p* = 0.000) and early infection (57.1% vs. 88.1%; *p* = 0.001). Age, ASA score, smoking and the surgical procedure lasting longer than 2.5 hours did not increase the risk for PJI caused by MDR- and XDR-GNB relative to the risk of PJI caused by other microorganisms (Table A1).

Variables identified as significant and clinically relevant in the univariate analysis were added to the multivariate model. In the multivariate model, the predisposing factors independently associated with PJI caused by MDR- and XDR-GNB were revision arthroplasty [PR: 1.7; 95% confidence interval (CI): 1.2–2.4; *p* = 0.002], previous orthopaedic infection (PR: 1.5; 95% CI: 1.1–2.1; *p* = 0.002), postoperative hematoma (PR: 2.6; 95% CI: 1.7–4.0; *p* < 0.001) and early infection (PR: 2.2; 95% CI: 1.4–3.5; *p* = 0.001) (Table 3).

No significant differences between groups were observed for the time between prosthesis placement and PJI diagnosis (*p* = 0.066) or the time between PJI diagnosis and treatment failure (*p* = 0.063) (Table A2). It is worth pointing out that the rate of PJI recurrence after treatment was lower among patients infected by MDR-/XDR-GNB than among those infected by other bacteria (4.1% and 6.1%, respectively). On the other hand, higher rates of death were observed in the MDR/XDR-GNB PJI group than in the ‘other bacteria’ PJI group (17.3% vs 5.1%; *p* = 0.038). Even though a comparison of the rate of treatment failure (recurrence/death) between groups (MDR- and XDR-GNB vs other microorganisms) showed no statistically significant difference (*p* = 0.264), a patient with PJI caused by XDR-GNB was 4.6 times more likely to progress to death than a patient with a PJI caused by other pathogens (odds ratio: 4.6; 95% CI: 1.4–15.7; *p* = 0.010). In contrast, progression to death was not more likely among patients with MDR-GNB PJIs than among those with PJIs caused by other microorganisms (odds ratio: 2.3; 95% IC: 0.6–7.9; *p* = 0.200). The risk of treatment failure was not significantly different between all GNB PJI cases and all GPC PJI cases (*p* = 0.516). Moreover, no significant differences in the outcome were observed when DAIR was performed versus the use of non-DAIR options (i.e., one-stage and two-stage exchange arthroplasty) (*p* = 0.842). 

**Figure 1:**
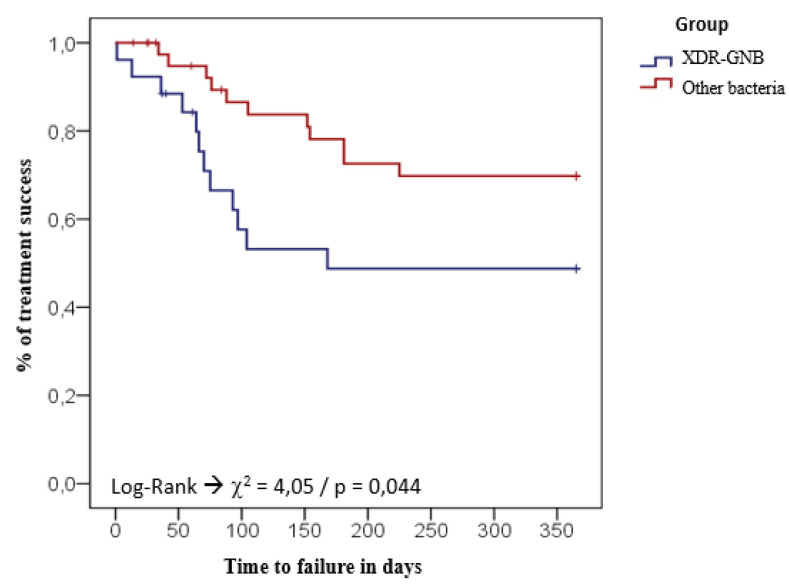
Kaplan–Meier survival curve for treatment failure (death/recurrence) considering PJIs caused by XDR-GNB and other bacteria.

**Figure 2:**
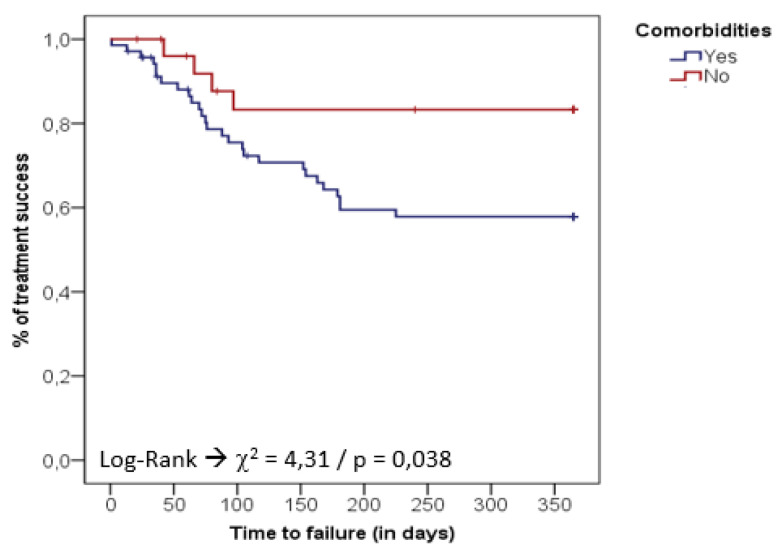
Kaplan–Meier survival curve for treatment failure (death/recurrence) among PJI patients with and without comorbidities.

However, according to the multivariate model, infections caused by XDR-GNB (PR: 2.3; 95% CI: 1.0–5.2; *p* = 0.044) and the presence of comorbidities (PR: 2.9; 95% CI: 1.0–8.4; *p* = 0.044) were strong predictive RFs independently associated with therapeutic failure (Table A3). The higher rates of treatment failure associated with XDR-GNB PJI and patients with comorbidities is best illustrated in Figure 1 and Figure 2. 

## 4. Discussion

In this study, revision arthroplasty, previous orthopaedic infection and postoperative hematoma were independently associated with the risk of developing MDR-GNB PJI. These RFs are well-known to be associated with any deep periprosthetic infection; however, the relevance of any infection-associated findings may vary depending upon the epidemiological context of an orthopaedic referral centre. At our centre, located in a large city in a developing country, the likelihood of nosocomial SSI caused by MDR-GNB is high. Early PJI was an additional RF identified for MDR-GNB PJI. We argue that the high selective pressure imposed by empirical and broad-spectrum antibiotic therapy, which is often prescribed for early PJI, may have had a major role not only in the higher prevalence of GNB-PJI, but also impacting on the lower rates GPC infections (20.2%), including the lack of CNS that was not identified in this study cohort.

MDR-GNB were identified in more than half of the bacterial populations isolated in this cohort study. Many other authors have reported that the likelihood of GNB as the etiological agent of SSI is greater in Latin America than in more developed regions [19,20]. In a Latin American surveillance study that included several medical centres, 12,811 bacterial species were isolated from several types of nosocomial infections, including SSIs; 44.5% of cases were identified as GNB infections with high rates of MDR [20].

Importantly, *A. baumannii* accounted for 33.7% of all GNB isolated in our study. Despite the implementation of many IC measures at our institution, the IC team has been unable to eradicate *A. baumannii* from the hospital environment. It is likely that this species has become an endemic pathogen responsible for nosocomial infections, including SSIs. A high prevalence of *A. baumannii* has been reported in other Latin American countries as well [21]. Some authors have suggested that the tropical climates and higher temperatures in Latin America may result in increased numbers of *Acinetobacter* spp. colonising the human skin, increasing the risk of nosocomial infections [22,23]. Typically, patients who undergo arthroplasty receive immediate postoperative care in the intensive care unit, which has been characterised as an institutional environment associated with an overwhelmingly high rate of *A. baumannii* colonisation relative to in other hospital units. This factor may increase the risk of *A. baumannii*–associated PJI.

Although previous authors have reported an increased risk for PJI with revision surgery [24,25,26], an association between revision surgery and GNB-MDR PJI has not been reported before now. In our study, a preceding PJI episode was associated with a 1.5-fold increase in the chance that the new PJI would be caused by MDR-GNB. This represents new and important epidemiological information. The occurrence of a previous PJI implies the prolonged use of combined and broad-spectrum antibiotics, and a direct association between prolonged antibiotic use and greater rates of MDR-GNB infections has been reported previously [27,28]. In a study by Benito et al. [29] of 2524 episodes of PJI, negative-coagulase staphylococci was identified as the most commonly identified causative pathogen, but GNB were more frequently identified in cases of early infection. Additionally, MDR-GNB accounted for nearly one-quarter of early PJIs and were identified three times more frequently in early infections than in late infections. The study by Benito et al. [29], conducted in Spain, was the first cohort study to identify the role played by MDR-GNB in early PJI episodes. Our data corroborate the association. 

The formation of hematoma or postoperative drainage for more than 2.5 days following arthroplasty has been identified previously as a predictor of wound infection for patients receiving hip and knee joint replacements [30]. In shoulder arthroplasty, an association between postoperative hematoma and subsequent PJI was documented by Cheung et al. [31] and Nagaya et al. [6]. Cheung et al. [31] identified various species of skin-associated microbiota in hematoma cultures, including *Cutibacterium acnes*, *Staphylococcus epidermidis* and other *Streptococcus* spp. The presence of postoperative hematoma in patients previously colonised with MDR-GNB may contribute to postarthroplasty infections, helping to explain our finding of postoperative hematoma as an independent risk factor for developing MDR-GNB PJI.

No significant differences in outcomes were observed when comparing infections caused by GNB and those caused by GPC. Uçkay and Bernard [32] reported similar success rates when treating PJIs caused by GPC or GNB. In contrast, several studies have linked GNB-associated PJI with high failure rates [16]. In our study, surgical options using DAIR had no impact on treatment outcomes, but XDR-PJI was independently associated with poor outcomes. In the study by Papadopoulos et al. [33], MDR- and XDR-GNB infections were associated with higher rates of therapeutic failure when DAIR was performed (52.2%) than when non-DAIR options were applied. Hiesh et al. [7] also reported worse outcomes when DAIR was the operative choice over non-DAIR options for GNB-associated PJI. Shohat et al. [17] reported higher failure rates for DAIR when treating PJIs caused by any MDR pathogens. However, in a study by Cobo et al. [34], the success rate of DAIR for early PJI was similar for GPC and GNB infections, with lower success rates reported for MRSA-affiliated PJI. 

The impact of XDR-GNB on poor outcomes may be associated with the general lack of antibiotic options for eradicating these bacteria, especially biofilm-acting quinolones. In our study, all XDR samples were quinolone resistant. Additionally, comorbidities had an independent negative effect on outcomes, increasing the likelihood of poor outcomes 2.9-fold. Multiple comorbidities may impact PJI outcomes in several ways, such as an increased likelihood of adverse events associated with prolonged and combined antibiotic therapy. Chronic comorbidities, such as kidney and liver failure, have also been associated with reduced immune responses against bacteria, allowing for the development of bone and biofilm infections.

The present study had several limitations. This study was performed as an observational, retrospective study conducted at a single centre offering special orthopaedic care to a regional population located in a major city in a developing country. Consequently, the results may not apply to other hospitals. Furthermore, the patients enrolled in the cohort were heterogeneous. Matching MDR-GNB with other bacteria may have biased our analysis, although multivariate analyses were performed to adjust for this variability. Also, bacterial identification and susceptibility tests were performed using nonautomated methods, and no molecular and genotypic analyses were performed to identify clonal variants or similar patterns of resistance mechanisms. Besides, all potential SCN growing in a single tissue sample culture were considered contaminants and were excluded from the analysis, which may have biased our results. In addition, the type of surgical approach for hip arthroplasty was not assessed. However, this study identified a large number of MDR-GNB infections, with a high frequency of XDR strains.

## 5. Conclusions

We found that revision arthroplasty, previous orthopaedic infection, postoperative hematoma and early PJI were predisposing RFs for MDR-GNB PJI. Infections caused by XDR-GNB and comorbidities were both associated with poor outcomes. Despite the limitations of our cohort study, these results may reflect the epidemiology of certain developing regions with weak antibiotic stewardship programs. The increasing prevalence of antimicrobial resistance among PJIs poses a challenge for practitioners.

## Figures and Tables

**Table 1:** Demographics and clinical characteristics of the study population.

Characteristics	Number of Patients No. (%) Total = 98
Age (years) (mean ± S.D.)	67.3 ± 13.2
P50 (P25–P75)	69.5 (58.7–77)
Age group	
up to 50	10 (1.2)
51–60	23 (23.5)
61–70	20 (20.4)
71–80	31 (31.6)
over 80	14 (14.3)
Time between prosthesis and diagnosis (days) P50 (P25–P 75)	32 (20–242)
**Variables related to the patient**	
Comorbidities (yes)	71 (72.4)
SAH ^a^	60 (61.2)
DM ^b^	20 (20.4)
Malnutrition	8 (8.2)
Anemia	2 (2.0)
Neoplasm	1 (1.0)
Lung disease	5 (5.1)
Metabolic syndrome	18 (18.4)
Cardiovascular disease	5 (5.1)
Other comorbidities ^c^	11 (11.3)
Previous use of an antimicrobial	37 (37.8)
**Variables related to the surgical procedure**	
Arthroplasty	
Total	75 (76.5)
Primary	56 (57.2)
Elective	59 (60.2)
Hip	82 (83.7)
DAIR ^d^	69 (70.4)
Procedure duration greater than 2.5 h	5 (5.1)
Blood transfusion	16 (16.3)
**Variables related to the postoperative period**	
Concomitant non-orthopedic infection	11 (11.2)
Previous ortopedic infection	19 (19.4)
Early infection	68 (69.4)
Sepsis	2 (2.0)

SAH ^a^: Systemic arterial hypertension; DM ^b^: Diabetes Mellitus; Other comorbidities ^c^: rheumatoid arthritis, hypothyroidism, hyperthyroidism, depression. DAIR ^d^: debridement and implant retention.

**Table 2:** Description of 104 microorganisms isolated from bone and soft tissue cultures of patients with PJI described in the study.

**Microbial isolates in 56 episodes of** **MDR/XDR GNB ^a^ PJI** **^b^**	**60 (100)**
*Acinetobacter baumannii*	31 (51.7)
*Enterobacter aerogenes*	8 (13.3)
*Klebsiella pneumoniae*	6 (10.0)
*Escherichia coli*	5 (8.3)
*Proteus mirabilis*	4 (6.7)
*Pseudomonas aeruginosa*	3 (5.0)
Others GNB-MDR	3 (5.0)
**Microbial isolates in 42 episodes of others bacterias PJI ^b^**	**44 (100)**
MSSA ^c^	16 (36.4)
*Pseudomonas aeruginosa*	6 (13.6)
MRSA ^d^	5 (11.4)
*Enterobacter aerogenes*	4 (9.1)
*Proteus mirabilis*	3 (6.8)
*Proteus vulgaris*	2 (4.5)
*Acinetobacter baumannii*	2 (4.5)
*Klebsiella pneumoniae*	2 (4.5)
*Morganella morganii*	1 (2.3)
*Enterobacter sakazakii*	1 (2.3)
*Enterobacter cloacae*	1 (2.3)
*Escherichia coli*	1 (2.3)

MDR/XDR GNB ^a^: Multidrug resistant/extensively drug-resistant, gram-negative bacteria; PJI ^b^; prosthetic joint infection; MSSA ^c^; Methicillin-sensitive *Staphylococcus aureus*, MRSA ^d^; Methicillin-resistant *Staphylococcus aureus*.

**Table 3:** Predisposing factors independently associated with MDR-GNB ^a^ PJI ^b^ in the multivariate analysis.

Variables	Prevalence Ratio 95% CI	*p*-Value ^c^
Revision arthroplasty	1.7 (1.2; 2.4)	0.002
Previous orthopedic infection	1.5 (1.1; 2.1)	0.020
Postoperative hematoma	2.6 (1.7; 4.0)	<0.001
Early infection	2.2 (1.4; 3.5)	0.001

MDR-GNB ^a^: Multidrug resistant gram-negative bacteria; PJI ^b^; prosthetic joint infection; *p*-values ^c^ < 0.05 were considered statistically significant.

## Data Availability

Study data supporting our results were registered at an open access virtual platform for registration of studies on humans performed in Brazil. The Brazilian Registry of Clinical Trials (ReBEC) http://www.ensaiosclinicos.gov.br/rg/RBR-6ft5yb/ Register Number: RBR-6ft5yb.

## Appendix A

**Presence of a major criteria**
1. Sinus tract with evidence of communication of the joint or visualization of the prosthesis
or
2. Identification of the same phenotypically similar pathogen in two or more different periprosthetic tissue samples or in joint fluid
**Presence of four or more minor criteria**
1. Presence of purulent periprosthetic secretion
2. Identification of acute inflammatory reaction in histopathologic tests of periprosthetic tissue
3. A single culture with the identification of a microorganism
4. High leukocyte cellularity in the synovial fluid
5. High percentage of neutrophils in the synovial fluid
6. Increased serum levels of C-reactive protein (CRP) or erythrocyte sedimentation rate (ESR)

## Appendix B

**Table A1:** Evaluation the influence of variables of interest on the outcome of PJI caused by MDR- GNB compared to other bacteria–univariate analysis.

Variables	PJI ^a^		
	MDR/XDR GNB ^b^	Other Bacteria	*p*-Value ^c^
	No. (%)	No. (%)	
	N = 56	N = 42	
**Demographic data**			
Males	29 (70.7)	12 (29.3)	0.021 *
Females	27 (47.4)	30 (52.6)	
Age (years)		F	
Mean ± Standard deviation	68.2 ± 13.8	66.0 ± 1.4	0.415 ***
Age group			
up to 50	7 (70.0)	3 (30.0)	0.126 **
51–60 years	13 (56.5)	10 (43.5)	
61–70 years	7 (35.0)	13 (65.0)	
71–80 years	18 (58.1)	13 (41.9)	
above 80 years	11 (78.6)	3 (21.4)	
**Variables related to the patient**			
Presence of comorbidities	41 (73.2)	30 (71.4)	0.845 *
SAH ^d^	33 (58.9)	27 (64.3)	0.590 *
DM ^e^	12 (21.4)	8 (19.0)	0.772 *
Malnutrition	7 (12.5)	1 (2.4)	0.133 **
Anemia	2 (3.6)	0 (0)	0.505 **
Neoplasm	0 (0)	1 (2.4)	0.429 **
Lung disease	0 (0)	5 (9)	0.013 **
Metabolic syndrome	6 (10.7)	12 (28.6)	0.024 *
Cardiovascular disease	4 (7.1)	1 (2.4)	0.388 **
Other comorbidities ^f^	4 (7.1)	7 (16.7)	0.197 **
Alcoholism	12 (21.4)	2 (4.8)	0.020 *
Smoking	9 (16.1)	4 (9.5)	0.344 *
ASA classification ^g^			
1	8 (14.3)	13 (31.0)	0.114 *
2	28 (50.0)	19 (45.2)	
3 or 4	20 (35.7)	10 (23.8)	
Previous orthopedic infection	20 (35.7)	0 (0)	0.000 *
**Previous use of antimicrobials (last three months)**			
Yes	31 (55.4)	6 (14.3)	0.000 *
Quinolones	12 (21.4)	3 (7.1)	0.052 *
β-Lactam Antibiotics	20 (35.7)	4 (9.5)	0.003 *
Antimicrobial combination	10 (17.9)	1 (2.4)	0.000 **
**Variables related to the surgical procedure**			
Arthroplasty			
Total	41 (73.2)	34 (81.0)	0.371 *
Revision	66.1 (37)	11.9 (5)	0.000 *
Non-elective	31 (55.4)	8 (19.0)	0.000 *
Hip	56 (100)	26 (61.9)	0.000 *
Duration of the procedure > 2.5 h	8 (14.3)	7 (16.7)	0.746 *
Blood transfusion	16 (28.6)	3 (7.1)	0.008 *
**Variables related to the postoperative period**			
Concomitant non-orthopedic infection	11 (19.6)	2 (4.8)	0.032 *
Previous orthopedic infection	20 (35.7)	0 (0)	0.000 *
Polymicrobial infection	4 (7.1)	4 (9.5)	0.721 **
Early infection	32 (57.1)	37 (88.1)	0.001 *
Postoperative hematoma	29 (51.8)	1 (2.4)	0.000 *
Sepsis associated with infection	2 (3.6)	0 (0)	0.505 **

PJI ^a^: Prosthetic Joints Infections; MDR/XDR GNB ^b^: Multidrug resistant/extensively drug-resistant, gram-negative bacteria; ^c^ *p*-values : <0.05 were considered statistically significant; SAH ^d^: Systemic arterial hypertension; DM ^e^: diabetes Mellitus; Other comorbidities ^f^: rheumatoid arthritis, hypothyroidism, hyperthyroidism, depression; ASA ^g^: American Anesthesiology Association. Significance probabilities refer to the Chi-squared test (*), Fisher’s exact test (**), and Student’s t-test (***).

**Table A2:** Comparative analysis between the two study groups regarding the time elapsed between prosthesis and diagnosis and time to therapeutic failure.

Variable	PJI by MDR-GNB	Descriptive Measures			*p* *
		Min-Max	Median (P_25_–P_75_)	Mean ± SD	
Time elapsed between prosthesis and diagnosis (days)	Yes	7.0–5.040.0	37.0 (20.3–472.5)	453.1 ± 934.7	0.066
	No	7.0–1.825.0	30.0 (20.0–39.3)	95.4 ± 285.4	
Time to failure (days)	Yes	1.0–179.0	68.0 (37.0–102.3)	77.1 ± 50.1	0.063
	No	34.0–225.0	105.0 (72.0–181.0)	119.1 ± 62.9	

* Significance probability refers to the Mann-Whitney test.

**Table A3:** Evaluation of the influence of variables of interest on the time to therapeutic failure–univariate and multivariate analysis.

Variables	Univariate Analysis	Prevalence Ratio 95% CI	*p*-Value ^a^
PJI ^b^ by GNB ^c^	0.087	-	-
PJI by MDR-GNB ^d^		1.0 (0.4; 2.5)	0.991
PJI by XDR-GNB ^e^		2.3 (1.0; 5.2)	0.044
DAIR ^f^ surgical strategy	0.842	-	-
Presence of comorbidities	0.038	2.9 (1.0; 8.4)	0.044

*p*-values ^a^ < 0.05 were considered statistically significant. PJI ^b^: Prosthetic Joints Infections; GNB ^c^: Gram-negative bacteria; MDR-GNB ^d^: multidrug-resistant, gram-negative bacteria; XDR-GNB ^e^: extensively-resistant, gram-negative bacteria; DAIR ^f^: debridement and implant retention. Significance probabilities in the univariate analysis refer to Log-Rank test. Significance probabilities in the multivariate analysis refer to Cox regression.

## References

[1] Kozak L.J., DeFrances C.J., Hall M.J. (2006). National Hospital Discharge Survey: 2004 Annual Summary with Detailed Diagnosis and Procedure Data. Vital. Health Stat..

[2] Corvec S., Portillo M.E., Pasticci B.M., Borens O., Trampuz A. (2012). Epidemiology and New Developments in the Diagnosis of Prosthetic Joint Infection. Int. J. Artif. Organs.

[3] Ong K.L., Kurtz S.M., Lau E., Bozic K.J., Berry D.J., Parvizi J. (2009). Prosthetic Joint Infection Risk After Total Hip Arthroplasty in the Medicare Population. J. Arthroplast..

[4] Martínez-Pastor J.C., Muñoz-Mahamud E., Vilchez F., García-Ramiro S., Bori G., Sierra J., Martínez J.A., Font L., Mensa J., Soriano A. (2009). Outcome of Acute Prosthetic Joint Infections Due to Gram-Negative Bacilli Treated with Open Debridement and Retention of the Prosthesis. Antimicrob. Agents Chemother..

[5] Tande A.J., Patel R. (2014). Prosthetic Joint Infection. Clin. Microbiol. Rev..

[6] Nagaya L.H., Salles M.J.C., Takikawa L.S.C., Fregoneze M., Doneux P., Da Silva L.A., Sella G.D.V., Miyazaki A.N., Checchia S.L. (2017). Infections after shoulder arthroplasty are correlated with higher anesthetic risk score: A case-control study in Brazil. Braz. J. Infect. Dis..

[7] Hsieh P., Lee M.S., Hsu K., Chang Y., Shih H., Ueng S.W. (2009). Gram-Negative Prosthetic Joint Infections: Risk Factors and Outcome of Treatment. Clin. Infect. Dis..

[8] Namba R.S., Inacio M.C., Paxton E.W. (2013). Risk Factors Associated with Deep Surgical Site Infections After Primary Total Knee Arthroplasty: An Analysis of 56,216 Knees. J. Bone Jt. Surg. Am. Vol..

[9] Malinzak R.A., Ritter M.A., Berend M.E., Meding J.B., Olberding E.M., Davis K.E. (2009). Morbidly Obese, Diabetic, Younger, and Unilateral Joint Arthroplasty Patients Have Elevated Total Joint Arthroplasty Infection Rates. J. Arthroplast..

[10] Zimmerli W., Trampuz A., Ochsner P.E. (2004). Prosthetic-Joint Infections. N. Engl. J. Med..

[11] Pradella J.G.D.P., Bovo M., Salles M.J.C., Klautau G.B., De Camargo O.A.P., Cury R.D.P.L. (2013). Infected primary knee arthroplasty: Risk factors for surgical treatment failure. Rev. Bras. Ortop..

[12] Benito N., Franco M., Ribera A., Soriano A., Rodriguez-Pardo D., Sorlí L., Fresco G., Fernández-Sampedro M., Del Toro M.D., Guío L. (2016). Time trends in the aetiology of prosthetic joint infections: A multicentre cohort study. Clin. Microbiol. Infect..

[13] Fantoni M., Borrè S., Rostagno R., Riccio G., Carrega G., Giovannenze F., Taccari F. (2019). Epidemiological and clinical features of prosthetic joint infections caused by gram-negative bacteria. Eur. Rev. Med. Pharmacol. Sci..

[14] Parvizi J., Zmistowski B., Berbari E.F., Bauer T.W., Springer B.D., Della Valle C.J., Garvin K.L., Mont M.A., Wongworawat M.D., Zalavras C.G. (2011). New Definition for Periprosthetic Joint Infection: From the Workgroup of the Musculoskeletal Infection Society. Clin. Orthop. Relat. Res..

[15] Magiorakos A.-P., Srinivasan A., Carey R.B., Carmeli Y., Falagas M.E., Giske C.G., Harbarth S., Hindler J.F., Kahlmeter G., Olsson-Liljequist B. (2012). Multidrug-resistant, extensively drug-resistant and pandrug-resistant bacteria: An international expert proposal for interim standard definitions for acquired resistance. Clin. Microbiol. Infect..

[16] Kandel C.E., Jenkinson R., Daneman N., Backstein D., Hansen B.E., Muller M.P., Katz K.C., Widdifield J., Bogoch E., Ward S. (2019). Predictors of Treatment Failure for Hip and Knee Prosthetic Joint Infections in the Setting of 1- and 2-Stage Exchange Arthroplasty: A Multicenter Retrospective Cohort. Open Forum Infect. Dis..

[17] Shohat N., Goswami K., Tan T.L., Fillingham Y., Parvizi J. (2019). Increased Failure after Irrigation and Debridement for Acute Hematogenous Periprosthetic Joint Infection. J. Bone Jt. Surg. Am. Vol..

[18] Leclercq R., Cantón R., Brown D., Giske C., Heisig P., MacGowan A., Mouton J., Nordmann P., Rodloff A., Rossolini G. (2013). EUCAST expert rules in antimicrobial susceptibility testing. Clin. Microbiol. Infect..

[19] Villegas M.V., Blanco M.G., Sifuentes-Osornio J., Rossi F. (2011). Increasing prevalence of extended-spectrum-betalactamase among Gram-negative bacilli in Latin America: 2008 update from the Study for Monitoring Antimicrobial Resistance Trends (SMART). Braz. J. Infect. Dis..

[20] Gales A.C., Castanheira M., Jones R.N., Sader H.S. (2012). Antimicrobial resistance among Gram-negative bacilli isolated from Latin America: Results from SENTRY Antimicrobial Surveillance Program (Latin America, 2008–2010). Diagn. Microbiol. Infect. Dis..

[21] Vega S., Dowzicky M.J. (2017). Antimicrobial susceptibility among Gram-positive and Gram-negative organisms collected from the Latin American region between 2004 and 2015 as part of the Tigecycline Evaluation and Surveillance Trial. Ann. Clin. Microbiol. Antimicrob..

[22] Kim Y.A., Kim J.J., Won D.J., Lee K. (2018). Seasonal and Temperature-Associated Increase in Community-Onset Acinetobacter baumannii Complex Colonization or Infection. Ann. Lab. Med..

[23] Perencevich E.N., McGregor J.C., Shardell M., Furuno J.P., Harris A.D., Morris J.G., Fisman D.N., Johnson J.A. (2008). Summer Peaks in the Incidences of Gram-Negative Bacterial Infection Among Hospitalized Patients. Infect. Control. Hosp. Epidemiol..

[24] Jacobs A.M.E., Bénard M., Meis J.F., Van Hellemondt G., Goosen J.H.M. (2017). The unsuspected prosthetic joint infection. Bone Jt. J..

[25] Hoell S., Moeller A., Gosheger G., Hardes J., Dieckmann R., Schulz D. (2016). Two-stage revision arthroplasty for periprosthetic joint infections: What is the value of cultures and white cell count in synovial fluid and CRP in serum before second stage reimplantation?. Arch. Orthop. Trauma Surg..

[26] Kunutsor S.K., Whitehouse M.R., Blom A.W., Beswick A.D., Team I. (2016). Patient-Related Risk Factors for Periprosthetic Joint Infection after Total Joint Arthroplasty: A Systematic Review and Meta-Analysis. PLoS ONE.

[27] Liu P., Li X., Luo M., Xu X., Su K., Chen S., Qing Y., Li Y., Qiu J. (2018). Risk Factors for Carbapenem-Resistant Klebsiella pneumoniaeInfection: A Meta-Analysis. Microb. Drug Resist..

[28] Raman G., Avendano E.E., Chan J., Merchant S., Puzniak L. (2018). Risk factors for hospitalized patients with resistant or multidrug-resistant Pseudomonas aeruginosa infections: A systematic review and meta-analysis. Antimicrob. Resist. Infect. Control..

[29] Benito N., Mur I., Ribera A., Soriano A., Rodríguez-Pardo D., Sorlí L., Cobo J., Fernández-Sampedro M., Del Toro M.D., Guío L. (2019). The Different Microbial Etiology of Prosthetic Joint Infections according to Route of Acquisition and Time after Prosthesis Implantation, Including the Role of Multidrug-Resistant Organisms. J. Clin. Med..

[30] Saleh K., Olson M., Resig S., Bershadsky B., Kuskowski M., Gioe T., Robinson H., Schmidt R., McElfresh E. (2002). Predictors of wound infection in hip and knee joint replacement: Results from a 20 year surveillance program. J. Orthop. Res..

[31] Cheung E.V., Sperling J.W., Cofield R.H. (2008). Infection Associated With Hematoma Formation After Shoulder Arthroplasty. Clin. Orthop. Relat. Res..

[32] Uçkay I., Bernard L. (2010). Gram-Negative versus Gram-Positive Prosthetic Joint Infections. Clin. Infect. Dis..

[33] Papadopoulos A., Ribera A., Mavrogenis A.F., Rodriguez-Pardo L., Bonnet E., Josésalles M., Del Toro M.D., Nguyen S., Blanco-García A., Skaliczki G. (2019). Corrigendum to “Multidrug-resistant and extensively drug-resistant Gram-negative prosthetic joint infections: Role of surgery and impact of colistin administration”. Int. J. Antimicrob. Agents.

[34] Cobo J., Miguel L.G.S., Euba G., Rodríguez D., García-Lechuz J., Riera M., Falgueras L., Palomino J., Benito N., Del Toro M. (2011). Early prosthetic joint infection: Outcomes with debridement and implant retention followed by antibiotic therapy. Clin. Microbiol. Infect..

